# A Fish-Specific Transposable Element Shapes the Repertoire of p53 Target Genes in Zebrafish

**DOI:** 10.1371/journal.pone.0046642

**Published:** 2012-10-31

**Authors:** Lucia Micale, Maria Nicla Loviglio, Marta Manzoni, Carmela Fusco, Bartolomeo Augello, Eugenia Migliavacca, Grazia Cotugno, Eugenio Monti, Giuseppe Borsani, Alexandre Reymond, Giuseppe Merla

**Affiliations:** 1 Medical Genetics Unit, IRCCS “Casa Sollievo della Sofferenza”, San Giovanni Rotondo, Italy; 2 Center for Integrative Genomics, University of Lausanne, Lausanne, Switzerland; 3 Department of Biomedical Science and Biotechnology, University of Brescia, Brescia, Italy; INRA, France

## Abstract

Transposable elements, as major components of most eukaryotic organisms' genomes, define their structural organization and plasticity. They supply host genomes with functional elements, for example, binding sites of the pleiotropic master transcription factor p53 were identified in LINE1, Alu and LTR repeats in the human genome. Similarly, in this report we reveal the role of zebrafish (*Danio rerio*) EnSpmN6_DR non-autonomous DNA transposon in shaping the repertoire of the p53 target genes. The multiple copies of EnSpmN6_DR and their embedded p53 responsive elements drive in several instances p53-dependent transcriptional modulation of the adjacent gene, whose human orthologs were frequently previously annotated as p53 targets. These transposons define predominantly a set of target genes whose human orthologs contribute to neuronal morphogenesis, axonogenesis, synaptic transmission and the regulation of programmed cell death. Consistent with these biological functions the orthologs of the EnSpmN6_DR-colonized loci are enriched for genes expressed in the amygdala, the hippocampus and the brain cortex. Our data pinpoint a remarkable example of convergent evolution: the exaptation of lineage-specific transposons to shape p53-regulated neuronal morphogenesis-related pathways in both a hominid and a teleost fish.

## Introduction

Transposable elements (TEs) represent the largest genomic component of most eukaryotic organisms. Their contribution to genome size reaches up to two third in vertebrate [1]–[3] invertebrate [4], and plant genomes [5], [6]. They play a central role in the structural organization and plasticity of genomes allowing the establishment of evolutionary new cellular functions (reviewed in [7]–[9]). The distinctive parasitic and selfish behavior of TEs provides a rational mechanism for their recruitment as genome building blocks. Genome sequencing projects and genome-wide functional assays showed that in several instances TEs facilitate the generation of new *cis*-regulatory elements by inserting themselves in close proximity to genes. They modulate and coordinate eukaryotic gene expression by supplying transcription factors binding sites [10], [11], transcription start sites [12], [13], and enhancer and silencers elements [14], [15]. Convergent domestication of transposons (i.e. adaptation of a transposon to be used for a host function) were recently uncovered. SINE family species-specific expansions remodel the repertoire of CTCF-binding sites in highly diverse mammalian lineages [16]. Likewise the recruitment of different and unrelated transposable elements control the extrapituitary expression of Prolactin in various mammal lineages [17]. The third example might stem from a predisposition of *pogo*-like transposases to encode centromeric proteins. Members of this family of transposons were tamed in mammalian and fission yeast genomes to encode centromere-associated protein B (CENP-B) and CENP-B-like proteins, respectively [18]. These examples of convergent evolution illustrate well previous findings of the ENCODE consortium that many functional elements are seemingly unconstrained constituting a “warehouse” for natural selection.

The co-option of TEs sequences to serve cellular function different from the role they were originally evolved for [19] is a remarkable molecular case of “exaptation” [20]. In this report, we describe the presence of functional binding sites (a.k.a responsive elements) for the p53 tumor suppressor protein, a key regulator of genes involved in cell-cycle progression, apoptosis, DNA repair, and cell senescence [21], [22], in a zebrafish-specific class of mobile elements named EnSpmN6_DR transposons. The multiple copies of these DNA transposable elements create an extensive transcriptional network of co-regulated genes. As primate-specific LTR class I endogenous retrovirus retroelements and Alu repeats were previously shown to concurrently provide a repertoire of responsive elements for p53 [23]–[25], our data pinpoint an interesting example of convergent evolution through exaptation of lineage-specific mobile elements.

## Results

### Zebrafish trim8a and trim8b

The tripartite motif proteins (TRIM) contain a motif composed of a RING finger structure, two B-box domains and a coiled-coil region followed by a more variable C-terminal portion [26], [27]. They are widely distributed in metazoans [28] and play important roles in biological processes as diverse as apoptosis, cell proliferation and viral defense [28], [29]. Recent studies have partially catalogued the repertoire of zebra- and pufferfish TRIM genes. They showed that teleosts contain a lineage specific subset of TRIM genes named finTRIM (fish novel TRIM genes) possibly involved in innate immunity [30]–[32].

We recently characterized an active p53 responsive element, composed of three decamers, in the first intron of the human *TRIM8* gene, a member of the tripartite motif protein family [33], [34]. To isolate the ortholog(s) of *TRIM8* in zebrafish we carried out a genome wide search using the sequences of the human TRIM8 polypeptide as query (NP_112174) using both the TBLASTN and BLAT algorithms *vs.* April 2007 (Zv7) and July 2010 (Zv9) *Danio rerio* assembly of genomic sequences. We identified two putative paralogous genes, which we named *trim8a* and *trim8b* mapping to chromosome 13 and 12, respectively. We then used the 27 and 40 ESTs deposited in dbEST (http://www.ncbi.nlm.nih.gov/dbEST/) overlapping *trim8a* and *trim8b*, respectively, to assemble putative transcripts that corresponded to the Zv9 models of these genes. The *trim8a* model (Zv9) spans 2081 bp and five exons encoding a 368 residues protein (ENSDARG00000090512), while the *trim8b* putative transcript is 3589 bp and six exons long (ENSDART00000085888; Zv7, Zv8 and Zv9 assemblies). It encodes a 564 residues protein (ENSDARP00000080323). To validate these models (especially as the Zv7 model of *trim8a* was different) we amplified by RT-PCR the open reading frames (ORF) of the *trim8a* and *trim8b* predicted cDNAs using total RNA from zebrafish embryos (of note all primers were designed using the Zv7 assembly), cloned them in a pcDNA3-HA vector and sequenced them for confirmation. The features of *trim8b* were corroborated, whereas we identified a longer *trim8a* ORF. It spans 1683 bp over six exons and encodes a peptide of 560 amino acid residues corresponding to the Zv7 model. Thus the two zebrafish *trim8* genes and their human counterpart have all maintained the same genomic structure (**Figure S1A**). The cDNAs corresponding to *trim8a* and *trim8b* are deposited in GenBank under the accession numbers JX266663-4.

We analyzed the genomic region surrounding the *TRIM8* genes in human, amphibian and zebrafish. A high degree of synteny was found between human and *Xenopus*, while a partial synteny was observed between the human gene and both *trim8a* and *trim8b* of *Danio rerio* (**Figure S1B**). We then explored the syntenic regions of these two paralogous genes using Genomicus [35] in a set of teleosts. Like zebrafish, the genomes of stickleback and medaka contain two *trim8* genes, whereas only the ortholog of *trim8b* can be identified in fugu and tetraodon (**Figure S2**). The numbers of trim8 genes in the different species is further supported by blat analysis on the UCSG genome browser and with the genomic data deposited in Ensembl (http://www.ensembl.org/Multi/Search/Results?species=allidx=q=trim8). The alignment of human, mouse, chicken, zebrafish and fugu sequences using mVISTA revealed that no conserved non-coding sequences shared among vertebrate genomes could be found within or in the vicinity of *TRIM8* genes [36], [37].

The multiple sequence alignment presented in **Figure S1C** show the high degree of amino acid sequence identity between the human and zebrafish TRIM8 proteins. *Danio rerio* Trim8a is 66% identical and 90% similar to human TRIM8, whereas Trim8b shows 67% identity and 87% similarity, respectively. The level of identity is particularly high in the first half of these proteins, a region that contains the tripartite motif [26], and in their C-terminal proline-rich region. The zebrafish Trim8 proteins show 72% of identity and 74% of similarity between them. Emblematic of TRIM proteins, TRIM8, Trim8a and Trim8b contain the canonical tripartite motif followed by a C-terminal portion, containing an uncharacterized sequence with structural similarity to synaptonemal complex protein 1 (SCP-1) and chromosome segregation protein-associated domains.

We then assessed the subcellular localization of TRIM8 proteins in human HeLa cells transfected with vectors transiently expressing EGFP-tagged zebrafish Trim8a, zebrafish Trim8b and human TRIM8. Both Trim8a and Trim8b proteins localize into discrete nuclear structures heterogeneous in size and shape, similarly to the nuclear structures described for the human TRIM8 protein [26](**Figure S3**). *In silico* analysis of Trim8a amino-acidic sequence predicted the presence of a nuclear localization signal (NLS) encompassing a KKEK signal conserved in human (**Figure S1C**). Consistently, the deletion of the C-terminal domain harboring the NLS induces the delocalization of the mutant protein into discrete cytoplasmic structures confirming that Trim8a C-terminal end is necessary for the proper nuclear localization of the protein (**Figure S3**), as observed for the human TRIM8ΔCter mutant [26]

### Functional p53 responsive elements map within a zebrafish-specific non-autonomous transposon

**Figure 1:**
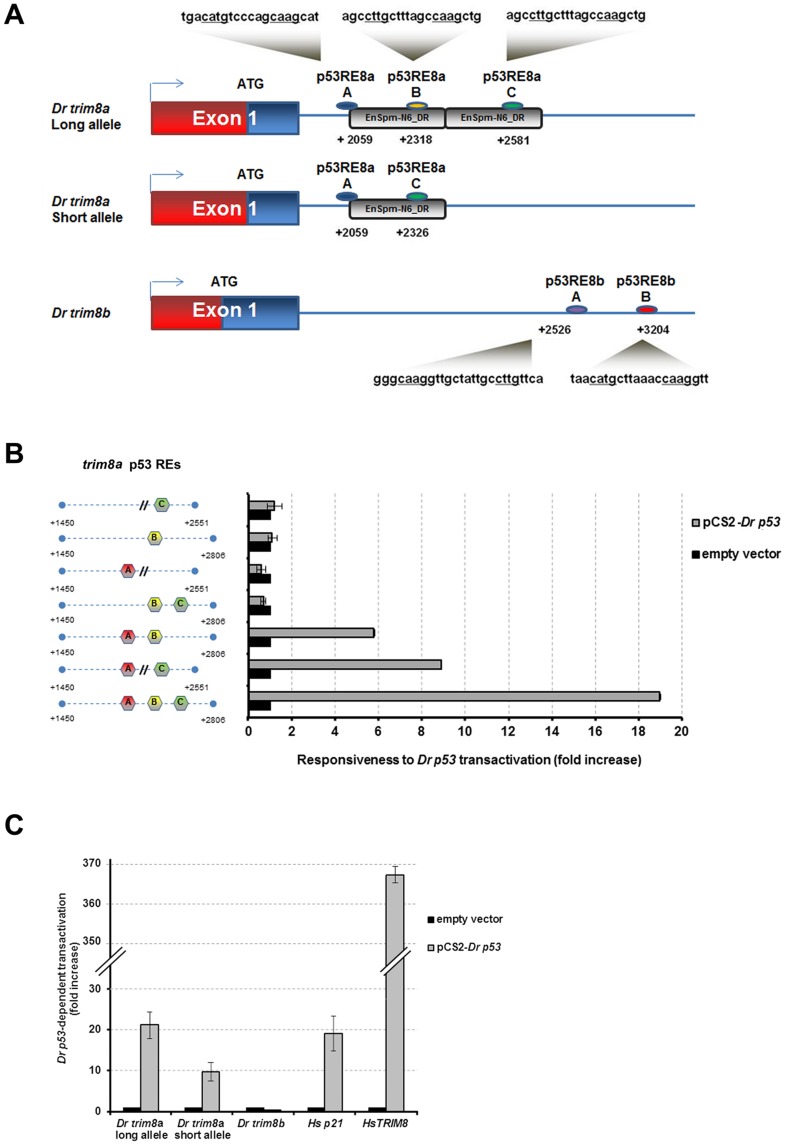
Functional p53 responsive elements map within the zebrafish-specific EnSpm-N6_DR transposon. (**A**) Predicted p53 responsive elements (REs) and their respective sequences in the first intron of *Danio rerio trim8a* long allele, *trim8a* short allele and *trim8b*. The indicated positions for REs are calculated from the annotated transcription start site. (**B**) Responsiveness to *Danio rerio* p53 transactivation of the first intron of *trim8a* engineered to contain different combinations of REs. The assessed mutant constructs are schematically represented on the left. (**C**) Zebrafish p53-dependent transactivation assessment in luciferase reporter assays of *Danio rerio trim8a* long allele, *trim8a* short allele, *trim8b* and *Homo sapiens p21* and *TRIM8*.

Upon screening of the promoter and intronic sequences of *trim8a* and *trim8b*, we identified three and two putative p53 responsive elements (REs) [38]–[40] in their first intron, respectively (**Figure 1A**). The two distal *trim8a* identical putative p53 REs (REs B and C) are embedded within two contiguous and incomplete copies of EnSpm-N6_DR, a 346 bp zebrafish-specific non-autonomous DNA transposon [41]. We found no significant similarity to EnSpm-N6_DR sequences in the genomes of medaka (*Oryzias latipes*), fugu (*Takifugu rubripes*), tetraodon (*Tetraodon nigroviridis*) and stickleback (*Gasterosteus aculeatus*) demonstrating that this element is lineage specific. Each EnSpm-N6_DR copy harbors two putative p53 binding sites, each composed of two decameric half-sites (**Figure S4**). They are specific to EnSpm-N6_DR transposons as other EnSpm non-autonomous DNA elements such as EnSpm-N1_DR, EnSpm-N4_DR and EnSpm-N7_DR do not contain p53 REs. Using bioinformatics tools we identified 210 copies of the EnSpm-N6_DR transposon within 196 loci in the zebrafish genome (**Table S1**; see Methods). EnSpm-N6_DR copies and its embedded p53 REs show approximately 90% and 93% of sequence identity, respectively (**Figure S5**). Of note the EnSpm-N6_DR transposon shows a tendency to insert closer to zebrafish genes, with a significant proportion of the TEs located inside the genes (*P* = 0.001853) when compared to other EnSpm non-autonomous DNA elements such as EnSpm-N1_DR, EnSpm-N4_DR and EnSpm-N7_DR (**Figure S6** and methods). Consistent with studies on human p53 REs, none were found in coding exons [42]. In 49% of the cases one or more additional putative p53 REs are mapping within 1 kb of the TE REs (e.g. RE A in *trim8a*, **Figure 1A**). We assessed whether *in silico* predicted REs drive p53-mediated transactivation or repression by using luciferase reporter assays. Short genomic regions containing the REs were inserted in plasmids containing the luciferase reporter gene, and transfected into the human p53-null H1299 cell line along with vectors expressing wild-type zebrafish or human p53, a amino-truncated zebrafish p53 that lacks both the Mdm2-interacting motif and the transcription activation domain (Δ1-113 *Dr*p53) or an empty vector (see methods for details). Both human and zebrafish wild-type p53 proteins' responsiveness were tested to confirm the reproducibility of the data in a mammalian culture system. Similarly, we tested the responsiveness of the *trim8* paralogs to p53. We found that the first intronic region of *trim8a* that encompass three predicted REs, two of them in the EnSpm-N6_DR transposable element (**Figure 1A**), is functionally activated by both zebrafish and human p53, but is unresponsive to Δ1-113 *Dr*p53 (**Figure 1C** **; Figure S7A–B**). In contrast the first intronic region of *trim8b*, which carries two p53 binding sequences not located into or nearby a transposon (**Figure 1A**) was not activated by p53 (**Figure 1C** **; Figure S7A–B**). During the cloning process we identified an allelic variant of the first intron of *trim8a* with a 255 bp deletion that encompass the second (B) transposon-embedded p53 RE (**Figure 1A**) in 45% of chromosomes (9 alleles with and 11 without the deletion in 10 investigated fishes). This “short allele” with only two REs activates transcription of the luciferase reporter 10 fold more than the empty vector, but about 2 fold less than the “long allele” that carries all three REs (**Figure 1C** **; Figure S7A–B**). Co-transfection of a cocktail of the full-length *Dr*p53 and Δ1-113 *Dr*p53 constructs reduced luciferase activity for the “long” and “short allele”, when compared to transfection with full-length *Dr*p53 protein only (**Figure S7B**consistent with the dominant negative activity of the truncated protein [43]. We then generated mutant constructs with only one or a combination of two *trim8a* REs and determined that the presence of the first RE (A) with one of the transposon-embedded REs (B or C) was necessary and sufficient to drive transactivation of the reporter gene (**Figure 1B**). To confirm these results *in vivo* we assessed expression of *trim8a* and *trim8b* in zebrafish embryos incubated for 16 hours in presence of 50 µM R-roscovitine, a cyclin-dependent kinase inhibitor that can efficiently stabilize and activate nuclear p53 in human and zebrafish cells [44], [45]. We observed an approximately 2 fold increase in *trim8a* mRNA levels upon exposure of 54 hpf-embryos to R-roscovitine, while no effect was detected on *trim8b* transcript (**Figure S7C**), confirming *trim8a* responsiveness to p53. Together these data indicate that only *trim8a* is, like its human counterpart, a p53 target gene.

**Figure 2:**
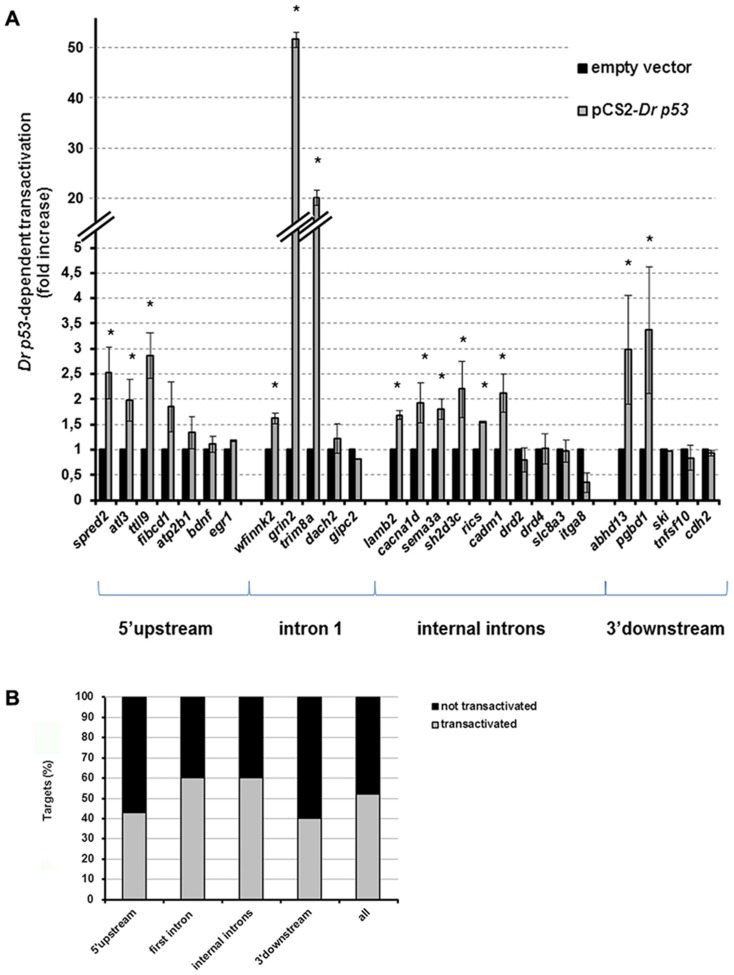
Transposon-embedded p53REs show p53-mediated transactivation. (**A**) Zebrafish p53-dependent transactivation assessment in luciferase reporter assays of 27 sequences containing EnSpm-N6_DR elements and mapping in close proximity to the indicated genes. Tested sequences are detailed in **Supplementary Table S2**, They are categorized by mapping location within gene loci, i.e. 5′, 3′, within the first or other more downstream exons. The names of the human orthologs are used for simplicity. Note that *atp2b1*, *cacna1d*, *dach2*, *drd2*, *drd4*, *fibcd1*, *grin2a*, *itga8*, *lamb2*, *pgbd1*, *rics*, *sema3a*, *sh2d3c*, *ski*, *tnfsf10*, *ttll9* and *wfikkn2* correspond to zebrafish *atp2b1a*, *cacna1da*, *dacha*, *drd4-rs*, *dkeyp-51b7.3*, *grin2aa*, *zgc:172265*, *lamb2l*, *si:ch73-353p21.4*, *dkey-269g4.4*, *sema3aa*, *sh2d3ca*, *skia*, *tnfsf10l4*, *si:dkey-211h10.2* and LOC564992, respectively. The percentage of transactivated targets per mapping class is shown in panel (**B**).

We further explored if the EnSpm-N6_DR element was associated with p53-mediated transactivation by testing the p53 responsiveness of 26 other genomic sequences in luciferase assays. These sequences contain one or two copies of the transposable element and additional non-transposon embedded p53REs. They originate from upstream/promoter, first intron, internal introns and downstream sequences of putative target genes (**Table S2** and methods). The sequence of the assessed TEs are deposited in GenBank under the accession numbers JX266665-693. A large proportion of the tested EnSpm-N6_DR elements and their adjacent sequences (14 out of 27, 52%) were responsive to *Dr*p53 co-transfection (**Figure 2**). We found no correlation between *Dr*p53-mediated transactivation and the position of the transposon within the putative target gene, as all tested classes of genomic sequences showed similar proportion of positive results (43% of tested promoter sequences (n = 7), 60% of first intron sequences (5), 60% of internal introns sequences (10) and 40% of downstream sequences (5); **Figure 2B**). A higher fraction of the sequences with putative supplementary non-transposon embedded p53REs showed p53-mediated transactivation (11 out of 18, 61%; e.g. *trim8a*, see above). The presence of these additional REs was no necessary, however, as some sequences such as *spred2*, *cacna1d* and *cadm1* with only EnSpm-N6_DR-embedded REs were responsive to p53 (**Figure 2A**).

### EnSpm-N6_DR-invaded genes are enriched for neural developmental genes

**Figure 3:**
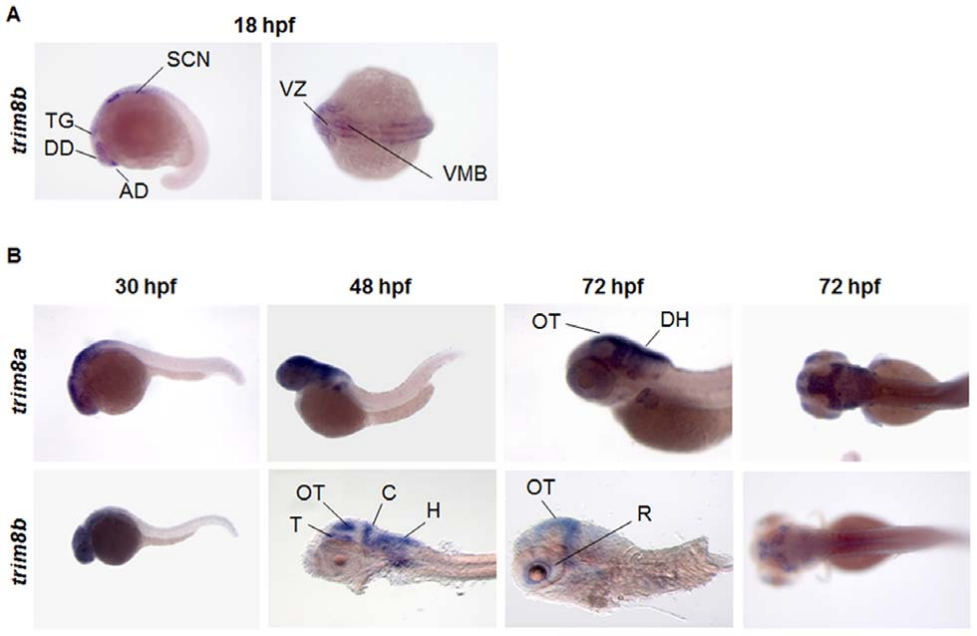
trim8a and trim8b expression in zebrafish embryos. (**A**) Expression pattern of *trim8b* at 18 hpf determined by whole mount ISH. Left panel: lateral view; right panel: dorsal view. Of note *trim8a* is not detected at this stage. (**B**) Comparison of the expression patterns of *trim8a* (top row) and *trim8b* (bottom row) after 30 to 72 hours of development. Embryos are in lateral (first three columns) or dorsal views (far right column). *Trim8b* expression at 48 hpf and 72 hpf (lateral view) is shown in de-yolked and flat mounted embryos (see Methods). Anterior to the left. AD, anterior diencephalon; C, cerebellum; DD, dorsal diencephalon; DH, dorsal hindbrain; H, hindbrain; OT, optic tectum; R, retina; SCN, ventral spinal chord neurons; T, tegmentum; TG, trigeminal ganglia; VMB ventro-lateral midbrain; VZ, brain ventricular zone.

To explore the function of EnSpm-N6_DR-invaded genes, we first examined by whole-mount *in situ* hybridization the expression patterns of *trim8a* and its paralog (**Figure 3**). Both genes are expressed in the Central Nervous System (CNS). While trim8a expression is not detectable early during zebrafish development, *trim8b* mRNA can be visualized during the segmentation period (**Figure 3A**). At 18 hpf *trim8b* is expressed in the brain rudiment: diencephalon, trigeminal ganglia, brain ventricular zone and ventro-lateral midbrain. At 30 and 48 hpf, *trim8a* shows diffuse expression throughout the CNS (**Figure 3B**, upper panels) that will restrict to more specific areas, such as the dorsal midbrain, specifically the tectum, the dorsal hindbrain and the retina during later stages of development (72 hpf). Its paralog, *trim8b*, stains diffusely the CNS region at 30 hpf (**Figure 3B**, lower panels). 18 hours afterwards it localizes to the mesencephalon, particularly the tectum dorsally and the midbrain tegmentum ventrally, the cerebellum and the dorsal hindbrain, before being restricted later in development (72 hpf) to the retina and optic tectum.

**Figure 4:**
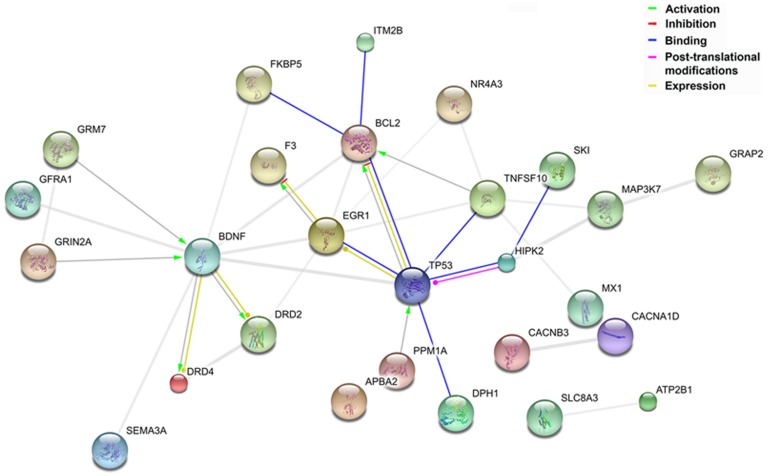
Protein subnetwork of human orthologs of genes parasitized by EnSpm-N6_DR elements in zebrafish classified by their involvement in specific developmental pathways, such as neurogenesis, synaptic transmission and regulation of programmed cell death. The network is visualized in STRING action view with lines and arrows of different colors indicating diverse types of interaction: binding (blue), activation (green), inhibition (red), post-translational modifications (violet) and co-expression (yellow). Of note the recently published interaction between TRIM8 and p53 [34] was added explicitly as it is not yet annotated within the STRING database.

Secondly, we analyzed the expression profiles of all the genes with EnSpm-N6_DR transposon insertions. Unfortunately the paucity of annotated expression in zebrafish prevented a meticulous analysis of the expression patterns as only 32 of the 193 zebrafish genes are annotated in Zf-Espresso (http://zf-espresso.tuebingen.mpg.de/, linked to the ZFIN Genomic Resources, http://zfin.org/); **Table S3**). However, as the patterns of expression of the zebrafish annotated genes is concordant to that of their mice and human orthologs, e.g. TRIM8 and trim8a and 8b (66% of concordance; **Table S4**) we can overcome this limitation by investigating the expression of their human orthologs identified using HomoloGene (http://www.ncbi.nlm.nih.gov/homologene; **Table S5**). We obtained data for 178 orthologs using UniProt (http://www.uniprot.org/). The orthologs of the parasitized genes are significantly enriched for genes expressed in adult and fetal brain, as well as specific brain structures, such as amygdala, hippocampus, and brain cortex (Benjamini's procedure for multiple testing corrections, corrected *P*<0.05; **Table S6**). Remarkably, 84% of these human genes were previously annotated as functionally validated or putative p53 targets in the p53FamTag database (149 out of 178 **Table S5** and methods). A proportion that should be compared to the about 1% of human protein coding genes (GENCODE v12; [46], [47]) cataloged as direct target of p53 [48]. A predicted functional classification of the encoded proteins is available for 172 of the 178 orthologs in DAVID (the Database for Annotation, Visualization and Integrated Discovery). Consistent with the expression data, the top five overrepresented Gene Ontology (GO) categories identified by these orthologs include neuron morphogenesis, axonogenesis, and the regulation of programmed cell death (**Table S7**), while KEGG (Kyoto Encyclopedia of Genes and Genomes) and Panther (Protein Analysis THrough Evolutionary Relationships) pinpoint enrichment of proteins involved in axon guidance (*P* = 0,0019; FDR<1%) and cell communication (GO: 0007154), respectively. Functional annotations as direct interaction, activation, repression or involvement in post-translational modifications are known for some of the human orthologs of EnSpm-N6_DR-colonized genes, which form a functional network with specific roles in neurodevelopmental pathways and apoptotic processes (**Figure 4** **and Figure S8**).

Although primate-specific TEs have been shown to contribute to the repertoire of p53 REs in humans [23]–[25], none of the analyses described in these references formally demonstrated that the p53-TE dependent genes were enriched for genes expressed in the CNS. Following a similar approach then the one used-above for the zebrafish orthologs, we show that the human genes neighboring p53 site-containing repeats described in [23] were significantly enriched for genes expressed in brain and brain structures (*P* = 0.0024), as well as for members of pathways involved in cell communication (GO:0007154) and adhesion (GO:0007155).

## Discussion

Recent studies that demonstrated the presence of p53 REs in human repeats and transposable elements have highlighted some of the mechanisms that introduce diversity in p53 responses. For example, a set of closely related families of long terminal repeat (LTR) elements (class I endogenous retroviruses) have promoted the spreading in the human genome of near-perfect p53 binding sites, which could be directly associated with p53-dependent transcriptional activation of the closest adjacent gene [23], [49]. Functional p53 REs embedded within primate-specific Alu elements and promoter sequence of the highly repetitive L1 elements (Long interspersed nuclear elements-1) were similarly shown to play an important role in shaping the p53 regulatory network [24], [25]. These cases of “exaptation” [20] of transposable elements (TEs) are not restricted to mammals, as in this work we identified p53 binding sequences in EnSpm-N6_DR elements, a zebrafish-specific non-autonomous transposon. Almost all of the 196 EnSpm-N6_DR insertions map in close proximity to genes, whose human orthologs were previously annotated as p53 targets (e.g. *TRIM8* [34], *BCL2* [50] and *EGR1* [51]). We assessed 27 of these genomic sequences and half of them drive p53-mediated activation confirming them as *bona fide* p53 REs. Few instances of convergent domestication of transposons (i.e. adaptation of a transposon to be used for a host function) were uncovered so far [16]–[18]. These examples of convergent evolution, as well as the one described in this report, illustrate well previous findings of the ENCODE consortium that many functional elements are seemingly unconstrained constituting a “warehouse” for natural selection [52]. Such turnover of regulatory elements was shown to be important to produce key phenotypic effect in multiple species [53], [54]. For example, the MER20 DNA transposon regulates progesterone- and cAMP-dependent gene expression through direct binding of factors essential for pregnancy in mammals [55].

The p53 transcription factor binds to specific responsive elements to regulate transcription of its target genes, thereby triggering cell-cycle arrest, promoting apoptosis, regulating differentiation or altering cellular lifespan in response to cellular stresses. Accumulating evidences demonstrate that p53 plays also a leading role in the regulation of neural stem cell proliferation and differentiation, as well as axon outgrowth and regeneration [56]–[58]. The domestication of EnSpm-N6_DR repeats and their embedded p53REs could be associated to the involvement of the targeted genes in shared pathways thus inducing these to act in concert via a p53-mediated mechanism of transcriptional regulation. We hypothesized that orthologous genes may have preserved ancestral regulation by p53. In such a scenario the insertion of EnSpmN6_DR elements might either not be disturbing the regulation already in place or be the driving force that triggered the establishment of a p53-centered network in zebrafish. We challenged this hypothesis by examining the annotation available for each human ortholog of zebrafish EnSpm-N6_DR-invaded gene through a network features enrichment tests. Consistent with the recently uncovered pivotal role of p53 in the CNS, these statistical analyses showed that the EnSpm-N6_DR-invaded genes are highly enriched in neuronal developmental pathways, such as neuron morphogenesis and axonogenesis, as well as programmed cell death. The functional networks formed by a subset of these genes (**Figure 4**) link the apoptosis regulators BCL2 [59] and HIPK2 [60] with EGR1, which controls the expression of genes involved in mitogenesis and differentiation [61]. While the activation of NMDA receptors, such as GRIN2A, and the release of the BDNF neutrophin contribute to synapse plasticity and development [62].

Overall, our results highlight that lineage-specific transposons were convergently domesticated in human and in zebrafish to establish networks of p53-regulated genes crucially involved in neuronal development. Our data further pinpoint how transposable element insertions shape genome functional evolution. Further studies are however warranted to understand if and how key cell regulators such as p53 are playing an active role in this “manipulation” of genomes.

## Methods

### Bioinformatic analyses

Nucleotide sequence assembly and editing were performed using the MacVector software programs (http://www.macvector.com/) DNA Strider and AutoAssembler (Perkin Elmer-Applied Biosystem), which is designed for assembly DNA sequences by importing text files and analysis files from Applied Biosystem automated sequencers. Zebrafish genomic sequences were analyzed using the University of California Santa Cruz (UCSC) Genome Browser (http://genome.ucsc.edu). Nucleotide sequences were compared to the non-redundant sequence databases present at the UCSC (University of California, Santa Cruz) using both BLAST and BLAT algorithms [63], [64]. Genomic sequence assemblies Zv9/danRer7-July 2010, GRCh37/hg19 - February 2009, NIG/UT MEDAKA1/oryLat2 - October 2005, JGI 4.0/fr2 - October 2004, Genoscope 8.0/tetNig2 - March 2007 and Broad/gasAcu1- February 2007 were used for zebrafish, human, medaka, fugu, tetraodon and stickleback, respectively. The online tool Genomicus (http://www.dyogen.ens.fr/genomicus-67.01/) was used to study conserved synteny for fish trim8 genes in teleosts.

The EnSpm-N6_DR non-autonomous transposable elements were identified by the RepeatMasker program (http://repeatmasker.genome.washington.edu) [65]. All EnSpm-N6_DR insertion sites were checked for the presence of coding sequences within 100 kb in each direction. To estimate if the transposable elements were significantly closer to genes (within 100 kb) we performed permutation tests (N = 10000) considering all Zebrafish RefSeq genes. To permute the elements we used shuffleBed from BEDtools version 2.10.1 [66].

Multiple sequence alignment was performed using the ClustalW algorithm (http://www.ebi.ac.uk/Tools/clustalw2/index.html) [67]. EnSpm-N6_DR neighboring regions (1 kb centromeric and telomeric from the transposon) were scanned using PatSearch (http://bighost.area.ba.cnr.it/BIG/PatSearch/) and the consensus sequence of the p53 Responsive Element (“canonical p53 REs” are composed of two decamers and a spacer as follows: RRRCWWGYYY…n…RRRCWWGYYY, in which R is a purine, Y is a pyrimidine, W is an A or T and the spacer is 0–13) [68].

We compare the localization of different subclasses of non-autonomous transposable DNA elements by calculating the distance to the closest transcript (**Figure S6**). Of note if the TE was intragenic the distance was considered 0. We then computed the proportions of TEs localized inside a gene for the four subclasses of assessed TEs and tested if these proportions were the same for all subclasses, p-value = 0.0018, X-squared = 14.9, df = 3 (**Figure S6**).

Human orthologs were searched for zebrafish genes carrying the transposable element by Ensembl (www.ensembl.org) and HomoloGene analysis (www.ncbi.nlm.nih.gov/homologene). p53FamTag database (http://p53famtag.ba.itb.cnr.it/), a resource of human direct p53 family target genes, was interrogated for the presence of putative or validated p53 REs in the human genes and for obtaining microarray experimental data.

ZfEspresso database (http://zf-espresso.tuebingen.mpg.de/) was employed to access zebrafish expression profiling data. DAVID tool (http://david.abcc.ncifcrf.gov) and Panther database for classification of genes and proteins (www.pantherdb.org) were used to functionally characterize human orthologs and zebrafish BLAT hits. STRING database (string-db.org/) of known and predicted proteins interactions was used to establish a protein network among human orthologs. We proceeded as follows: first, we identified functional modules using the whole list of human orthologs plus human p53, thus obtaining a global BLAT hits orthologous protein network; then, we analyzed the most significant nodes by assigning Gene Ontology (GO) terms to each modules. As a result, we found these genes fell into four significant functional networks. A merged and integrated network was obtained from two of them, comprising proteins with specific roles in neurodevelopmental pathways and apoptotic processes, respectively (**Figure 4**).

### Transient transfection and dual-luciferase reporter assay

We selected 27 sequences mapping close or within putative target genes, including *trim8a*, for responsiveness to *Dr*p53. We picked sequences with a high BLAT score and representative of all categories of genomic regions, i.e. upstream/promoter, first intron, internal introns and downstream sequences. EnSpm-N6_DR-containing genomic regions were amplified by polymerase chain reaction (PCR) using as a template zebrafish genomic DNA from a pool of fishes and cloned into pGL3-Basic vector (Promega, Madison, WI, USA). A complete list of primers is available on demand.

700 ng of the reporter construct, 5 ng of pSV-Renilla (pRL-SV40, Promega) and 100 ng of the indicated cDNAs expression construct (pCS2+HA-*Drp53*) or empty vector (pCS2+HA vector) were cotransfected into H1299 cells using FuGene HD Transfection Reagent (Roche). Cells were grown at 28°C, as *Danio rerio* p53 activity was shown to be labile at 37°C due to the presence of a threonine residue at position 128 (data not shown). 48 h after transfection, cells were assayed for both firefly and renilla luciferase activity using the Dual-GLO® Luciferase Assay System (Promega) using a Glomax 96 microplate luminometer. Firefly luciferase activity was normalized to *Renilla* luciferase activity for each transfected well. Values are the mean ± S.E.M. of three experimental replicates from two to four independent transfections.

### Roscovitine treatment and quantitative PCR

54 hpf-old zebrafish embryos were incubated in water- containing 50 µM R-roscovitine (Calbiochem) or 0.1% (v/v) DMSO for 16 hours at 28.5°C. Embryos were grown for 8 more hours in absence of roscovotine, harvested, washed in PBS-DEPC and cryoconserved at −80°C before performing the RNA extraction. The relative expression levels of *trim8a*, *trim8b* and *Dr*p21, a *Drp53* target gene, were examined by quantitative amplification with the following primer sets: *trim8a* 5′-TATGAAGAACACAAAGCCAGTGAAA-3′ and 5′-AACGCTGCCTGTGCATGA-3′, respectively as forward and reverse primers, *trim8b* 5′-CCACGCGGTGTGCGATA-3′ and 5′-ATCCGATCCTGCTGTTTAATCAG-3′, *Drp21* 5′-ACCCGTCCAGCTTCACACA-3′ and 5′-CTTCCACGAACGATGCTCTTC-3′. *DrEif1a* was used as a housekeeping gene and amplified by using the indicated forward 5′-CGATTCCACCGCATTTGTAGA-3′ and reverse 5′-CCACGTCGACTCCGGAAA-3′ primers. The reactions were run in triplicate in 10 µl of final volume with 10 ng of sample cDNA, 0.3 mM of each primer, and 1× Power SYBR Green PCR Master Mix (Applied Biosystems). Reactions were set up in a 384-well plate format with a Biomeck 2000 (Beckmann Coulter, Milan, Italy) and run in an ABI Prism7900HT (Applied Biosystems) with default amplification conditions. Raw Ct values were obtained using SDS 2.3 (Applied Biosystems). Calculations were carried out by the comparative Ct method.

### Fish breeding, embryo collection and whole mount in Situ Hybridization

Adult zebrafish were bred through natural crossings. Immediately after spawning, the fertilized eggs were harvested, washed and placed in 100-mm-diameter Petri dishes (Corning Life Sciences) in fish water [69]. The developing embryos were incubated at 28.5°C until use. Zebrafish embryos were fixed in 4% (w/v) paraformaldehyde/PBS overnight at 4°C, rinsed twice in PBS/1% Tween 20, then dehydrated in methanol and stored at −20°C until processing. Developmental stages of zebrafish embryos were expressed as hpf or dpf (hours or days post-fertilization respectively) at 28.5°C.

Single hybridizations and detections were carried out on wild-type embryos. Two distinct probes were prepared for each *trim8* paralog, one covering the entire coding region, and a second one corresponding to the more divergent C-terminal region. The observed expression patterns were entirely superimposable. Anti-sense and sense RNA probes were prepared by *in vitro* transcribing linearized cDNA clones or PCR products with T7, T3 or SP6 polymerase as indicated, using digoxigenin labeling mix (Roche). Stained embryos were transferred into 90% glycerol, or –where indicated- de-yolked and flat mounted in glycerol, in the middle of a bridged coverslip, covered with a top coverslip. Embryos were observed on a Leica MZ16F compound microscope, and acquired with a Leica DFC480 R2 digital camera and Leica Application Suite software Version 2.8.1.

## Supporting Information

Figure S1 **TRIM8 paralogous genes in fish.** (**A**) Schematic exon/intron organization of the human *TRIM8* (top) and zebrafish *trim8a* and *trim8b* genes. Exons (represented by rectangles) and introns (dashed line) are indicated with their respective length. UTRs are depicted in red, while ORF sequences are shown in grey (human) and blue (zebrafish). (**B**) Synteny of *TRIM8* loci in human, amphibian and zebrafish. (**C**) Protein sequences alignment showing the high degree of conservation of the amino acid sequences of HsTRIM8, DrTrim8a and DrTrim8b. The conserved residues of the RING, the B-box type 1 and the B-box type 2 are highlighted in yellow, green and blue, respectively. The Coiled-coil domain is underlined in black, proline-rich region residues are marked in red, while the nuclear localization signal is pinpointed by magenta asterisks.
(TIF)

Figure S2 **Trim8a and trim8b synteny in teleost species.** Graphical representation of conserved synteny around the *trim8a* and *trim8b* loci in teleosts generated using the Genomicus synteny browser. The figure is edited from the PhyloView display taking *trim8a* (**A**) and *trim8b* (**B**) as reference (both shown in light green in the center of the figures). Orthologs in different species are shown in matching colors, shaded genes correspond to genes that are not orthologous to any genes from the species used in the query. The synteny analysis combined with the analysis of UCSC and Ensemble genome browsers both indicate that in stickleback (*Gasterosteus aculeatus*) and medaka (*Oryzias latipes*) there are two *trim8* genes while in fugu (*Takifugu rubripes*) and in *Tetraodon nigroviridis* there is only one *trim8* ortholog. The presence of a single *trim8* gene in the two pufferfish species (*Takifugu rubripes* and *Tetraodon nigroviridis*) could be due either to the presence of gaps in the assembled genomes or to a selective gene loss possibly related to the extreme reduction in genome size so characteristic of that family.
(TIF)

Figure S3 **Subcellular localisation of TRIM8 proteins.** Subcellular localization of EGFP-tagged human TRIM8, zebrafish wild type and mutant (ΔC-terminus) Trim8a and zebrafish Trim8b in HeLa cells.
(TIF)

Figure S4 **EnSpm-N6_DR sequence.** Nucleotide sequence of 346 nucleotide long zebrafish-specific EnSpm-N6_DR non-autonomous transposon located in the first intron of the *trim8a* gene. The two overlapping p53 binding sites sequences predicted using the PatSearch algorithm are underlined in blue and red, while terminal inverted repeats are highlighted in yellow.
(TIF)

Figure S5 **Conservation of EnSpm-N6_DR.** Sequence alignment of the ten sequences with the best BLAT hit and mapping in close proximity to genes using the EnSpm-N6_DR transposon consensus as query sequence. Bases conserved in all ten sequences are in red. The predicted p53 REs are highlighted in purple in the consensus sequence (bottom line).
(TIF)

Figure S6 **Mapping positions of EnSpm-N transposons.** The stripcharts show the distribution of the distances between the TEs and the closest transcript, log10(distance+1), for four subclasses of non-autonomous transposable DNA elements. The yellow dots pipoint the median distance of the TEs to the closest transcript. Some horizontal jitter was added to improve the visual presentation of the plotted data. The numbers indicate the proportion (prop) of intergenic TEs for each subclass. The fractions of total genomic sequence distant more than 10e5, 10e4, 10e3, 10e2, 10 and 0 kb of a gene is reported on the y-axis, right hand side.
(TIF)

Figure S7 ***trim8a*** **is a p53 target gene in zebrafish.** (**A**) Human p53-dependent transactivation assessment in luciferase reporter assays of *Danio rerio trim8a* long allele, *trim8a* short allele, *trim8b* and *Homo sapiens p21* and *TRIM8* (**B**) Zebrafish p53-dependent transactivation assessment in luciferase reporter assays of *Danio rerio trim8a* long allele, *trim8a* short allele and *trim8b* upon transfection of full-length zebrafish p53 protein, a truncated form that lacks both the Mdm2-interacting motif and the transcription activation domain (Δ1-113 *Dr*p53) or co-transfection of this mutated form and full-length p53. (**C**) Relative expression levels of *Danio rerio p21*, *trim 8a* and *trim8b* mRNA in 54 hours old zebrafish embryos incubated for 16 hours in presence or absence of R-roscovitine, a p53 activator in human and zebrafish cells.
(TIF)

Figure S8 **Protein networks of human orthologs of genes parasitized by EnSpm-N6_DR elements.** (**A**) Global protein network of the human orthologs of genes colonized by EnSpm-N6_DR elements in zebrafish. (**B**) Additional protein subnetworks of these human orthologs. Asterisks' colors pinpoint the involvement of each gene to GO term-defined pathways. All subnetworks are visualized in STRING confidence view (the color saturation of the edges represents the confidence score of a functional association).
(TIF)

Table S1 **Complete list of zebrafish BLAT hits containing one or more EnSpm-N6_DR elements.** A complete list of BLAT hits, obtained using the zebrafish EnSpm-N6_DR transposable element as query vs the Zv9 (UCSC danRer7, Jul/2010) assembly of zebrafish genome, is reported, together with the list of genes whithin whom the transposon maps or flanking the transposon, with the relative distance.
(XLSX)

Table S2 **Putative p53 targets luciferase assay tested summary.**
(XLSX)

Table S3 **Expression data obtained by in situ experiments collected in ZfEspresso database for BLAT-list defined zebrafish genes.**
(XLSX)

Table S4 **Expression data obtained by in situ experiments collected in e-mouse atlas gene expression (EMAG,** http://www.emouseatlas.org/emage/home.php **) for those genes whose zebrafish orthologs are found in Zf-Espresso.**
(XLSX)

Table S5 **Human orthologs of zebrafish genes carrying the EnSpm-N6_DR transposable element.**
(XLSX)

Table S6 **Uptissue expression pattern of the human orthologs of the genes harboring the EnSpm-N6_DR transposable element.**
(XLSX)

Table S7 **DAVID functional annotation clustering of human orthologs.**
(XLSX)
