# Supplementary figures and images for: A Fish-Specific Transposable Element Shapes the Repertoire of p53 Target Genes in Zebrafish
Source: PLoS One. 2012 Oct 31;7(10):e46642. doi: 10.1371/journal.pone.0046642 (PMC3485254; doi:10.1371/journal.pone.0046642)

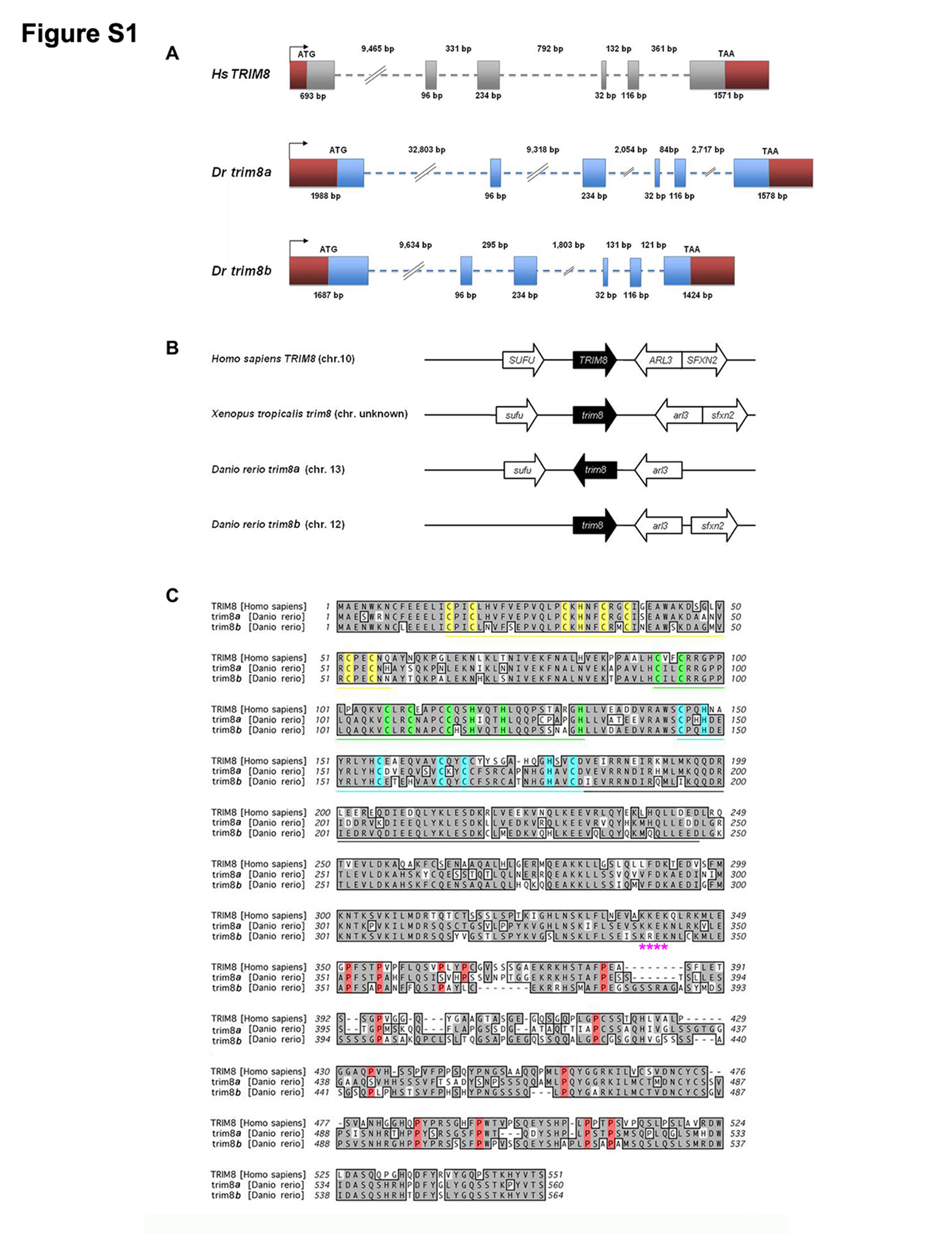

Supplement: Figure S1 — TRIM8 paralogous genes in fish. (A) Schematic exon/intron organization of the human TRIM8 (top) and zebrafish trim8a and trim8b genes. Exons (represented by rectangles) and introns (dashed line) are indicated with their respective length. UTRs are depicted in red, while ORF sequences are shown in grey (human) and blue (zebrafish). (B) Synteny of TRIM8 loci in human, amphibian and zebrafish. (C) Protein sequences alignment showing the high degree of conservation of the amino acid sequences of HsTRIM8, DrTrim8a and DrTrim8b. The conserved residues of the RING, the B-box type 1 and the B-box type 2 are highlighted in yellow, green and blue, respectively. The Coiled-coil domain is underlined in black, proline-rich region residues are marked in red, while the nuclear localization signal is pinpointed by magenta asterisks. (TIF) [file pone.0046642.s001.tif]

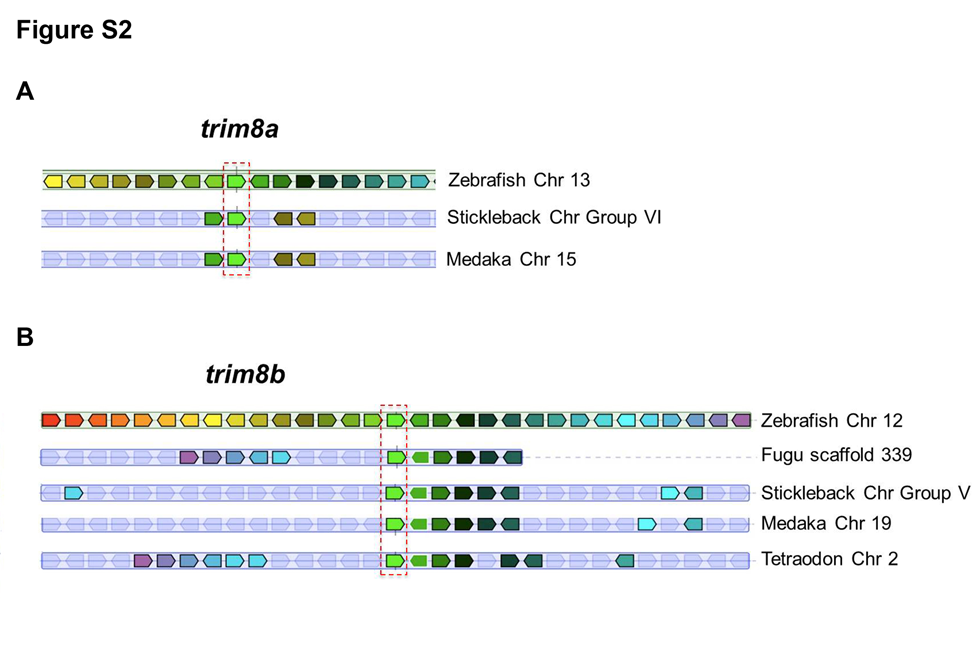

Supplement: Figure S2 — Trim8a and trim8b synteny in teleost species. Graphical representation of conserved synteny around the trim8a and trim8b loci in teleosts generated using the Genomicus synteny browser. The figure is edited from the PhyloView display taking trim8a (A) and trim8b (B) as reference (both shown in light green in the center of the figures). Orthologs in different species are shown in matching colors, shaded genes correspond to genes that are not orthologous to any genes from the species used in the query. The synteny analysis combined with the analysis of UCSC and Ensemble genome browsers both indicate that in stickleback (Gasterosteus aculeatus) and medaka (Oryzias latipes) there are two trim8 genes while in fugu (Takifugu rubripes) and in Tetraodon nigroviridis there is only one trim8 ortholog. The presence of a single trim8 gene in the two pufferfish species (Takifugu rubripes and Tetraodon nigroviridis) could be due either to the presence of gaps in the assembled genomes or to a selective gene loss possibly related to the extreme reduction in genome size so characteristic of that family. (TIF) [file pone.0046642.s002.tif]

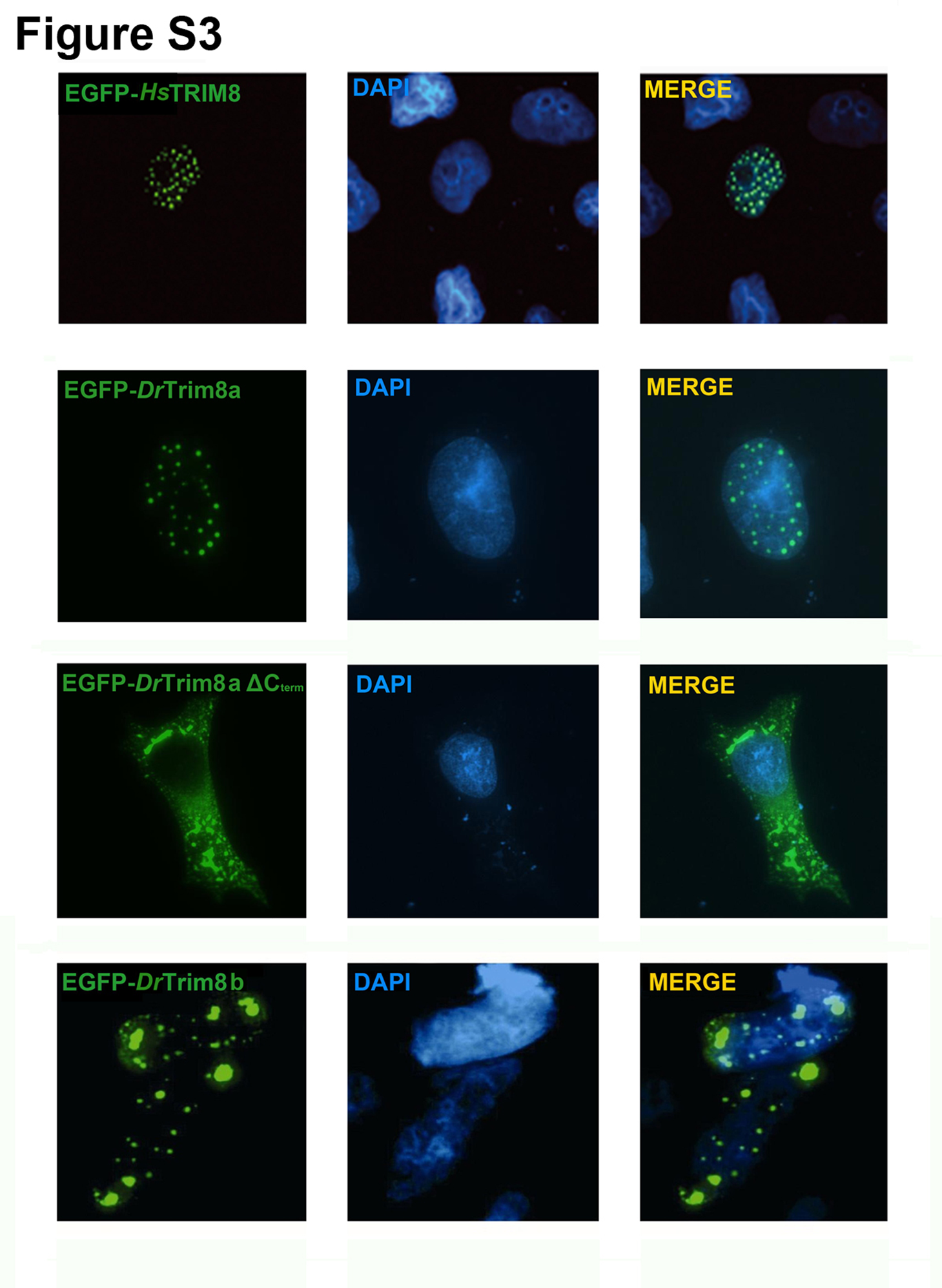

Supplement: Figure S3 — Subcellular localisation of TRIM8 proteins. Subcellular localization of EGFP-tagged human TRIM8, zebrafish wild type and mutant (ΔC-terminus) Trim8a and zebrafish Trim8b in HeLa cells. (TIF) [file pone.0046642.s003.tif]

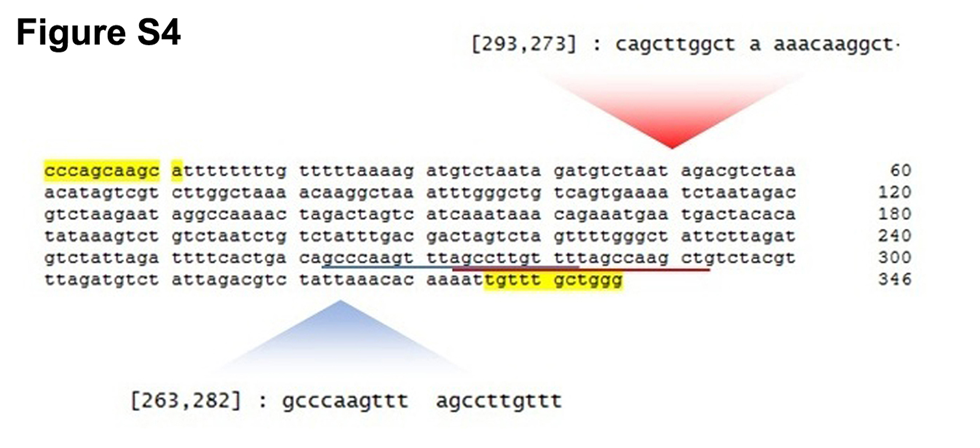

Supplement: Figure S4 — EnSpm-N6_DR sequence. Nucleotide sequence of 346 nucleotide long zebrafish-specific EnSpm-N6_DR non-autonomous transposon located in the first intron of the trim8a gene. The two overlapping p53 binding sites sequences predicted using the PatSearch algorithm are underlined in blue and red, while terminal inverted repeats are highlighted in yellow. (TIF) [file pone.0046642.s004.tif]

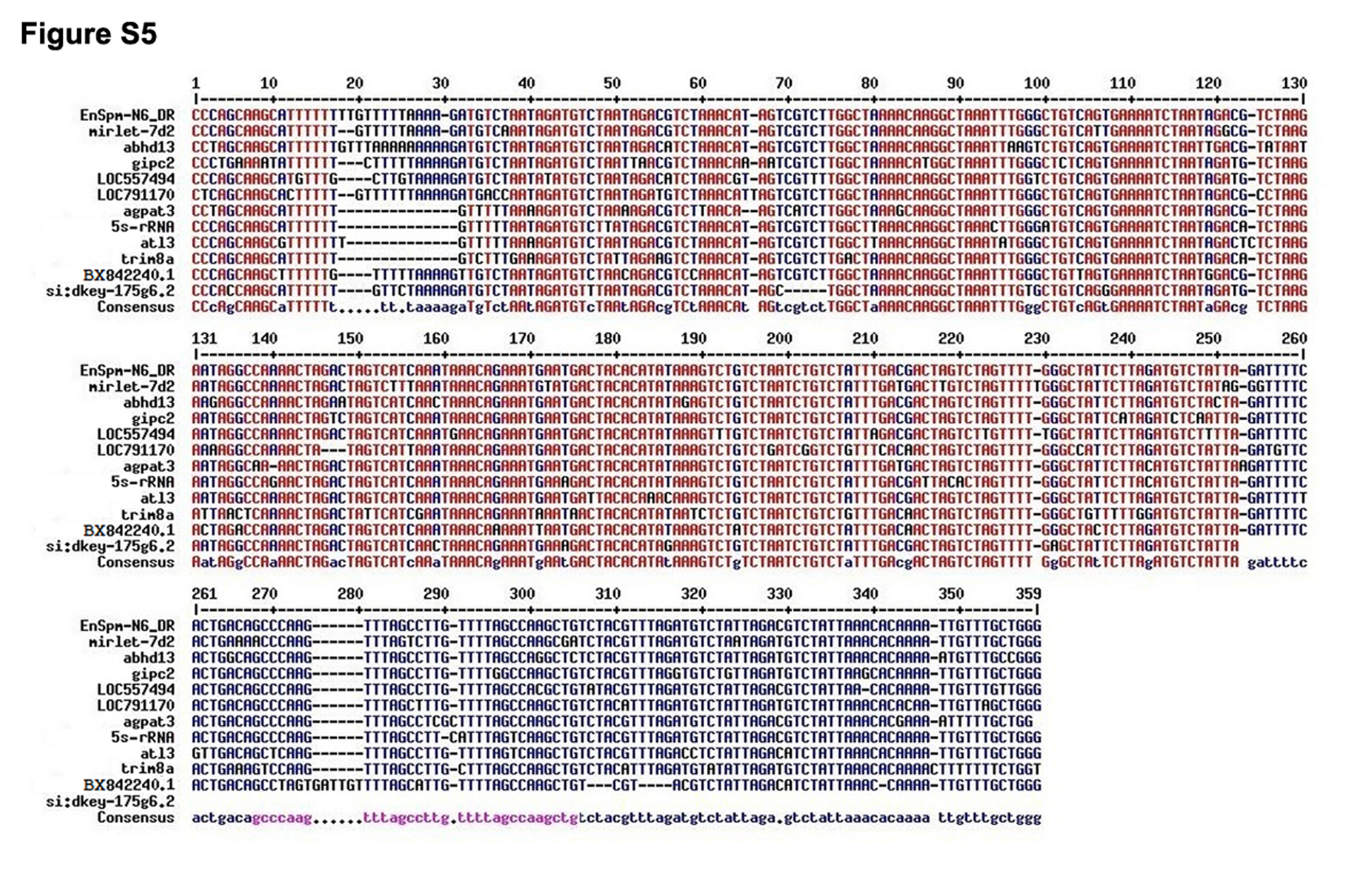

Supplement: Figure S5 — Conservation of EnSpm-N6_DR. Sequence alignment of the ten sequences with the best BLAT hit and mapping in close proximity to genes using the EnSpm-N6_DR transposon consensus as query sequence. Bases conserved in all ten sequences are in red. The predicted p53 REs are highlighted in purple in the consensus sequence (bottom line). (TIF) [file pone.0046642.s005.tif]

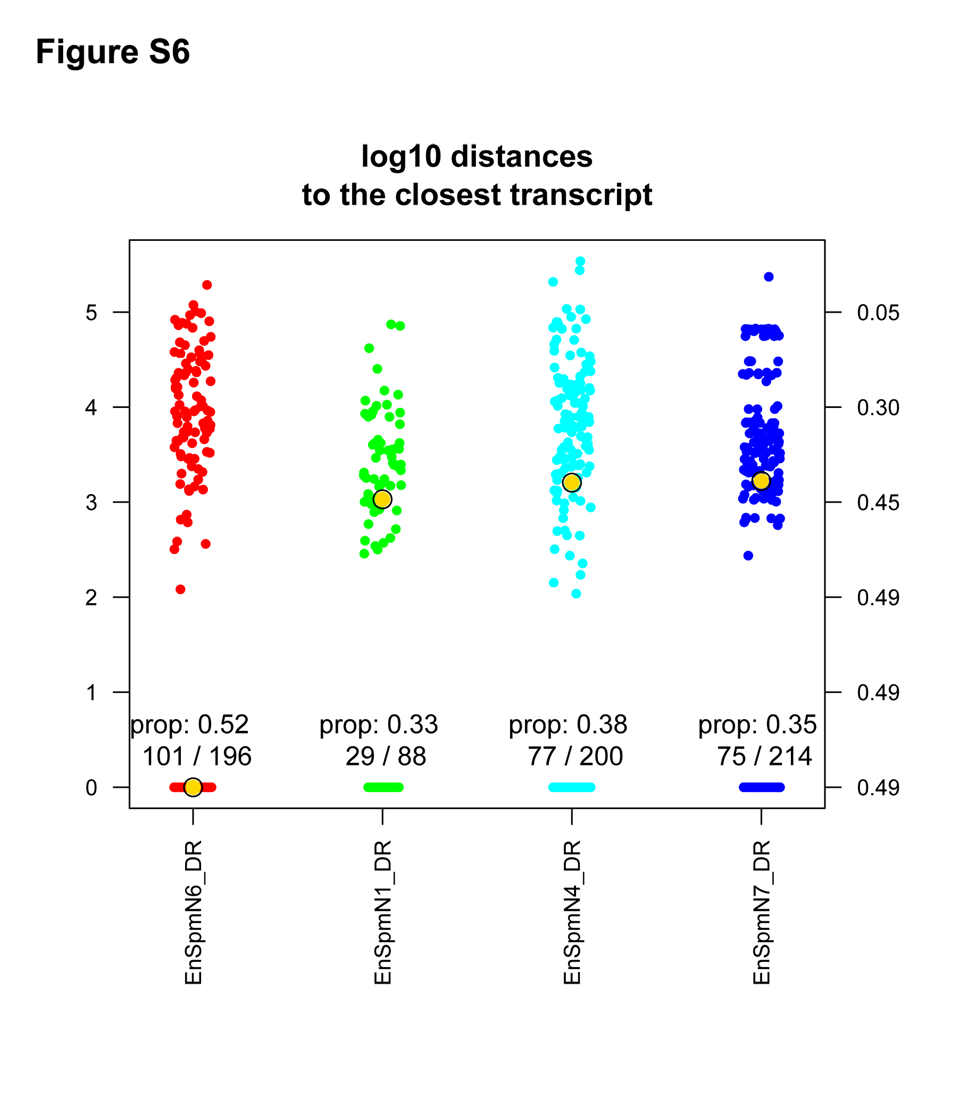

Supplement: Figure S6 — Mapping positions of EnSpm-N transposons. The stripcharts show the distribution of the distances between the TEs and the closest transcript, log10(distance+1), for four subclasses of non-autonomous transposable DNA elements. The yellow dots pipoint the median distance of the TEs to the closest transcript. Some horizontal jitter was added to improve the visual presentation of the plotted data. The numbers indicate the proportion (prop) of intergenic TEs for each subclass. The fractions of total genomic sequence distant more than 10e5, 10e4, 10e3, 10e2, 10 and 0 kb of a gene is reported on the y-axis, right hand side. (TIF) [file pone.0046642.s006.tif]

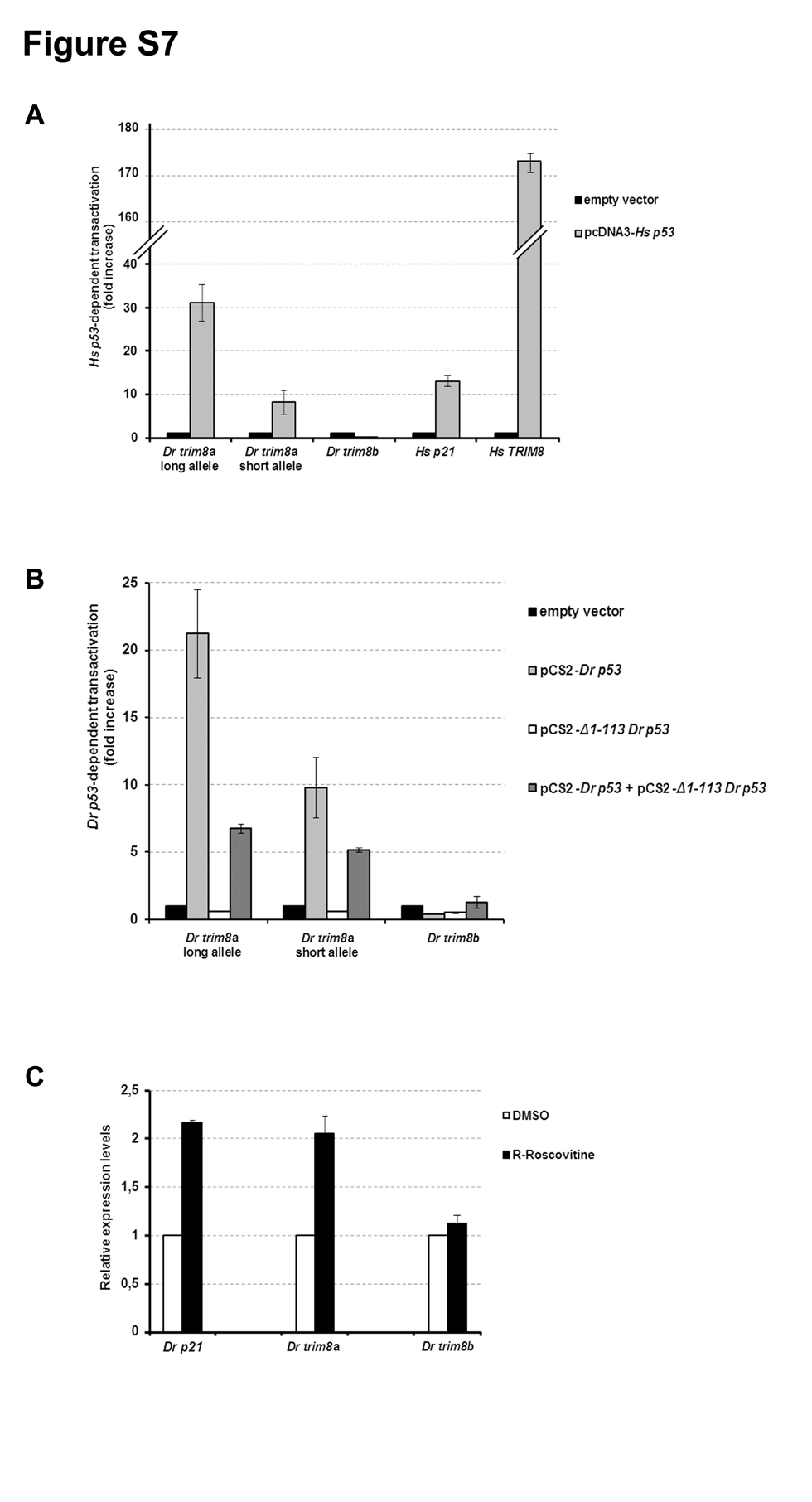

Supplement: Figure S7 — trim8a is a p53 target gene in zebrafish. (A) Human p53-dependent transactivation assessment in luciferase reporter assays of Danio rerio trim8a long allele, trim8a short allele, trim8b and Homo sapiens p21 and TRIM8 (B) Zebrafish p53-dependent transactivation assessment in luciferase reporter assays of Danio rerio trim8a long allele, trim8a short allele and trim8b upon transfection of full-length zebrafish p53 protein, a truncated form that lacks both the Mdm2-interacting motif and the transcription activation domain (Δ1-113 Drp53) or co-transfection of this mutated form and full-length p53. (C) Relative expression levels of Danio rerio p21, trim 8a and trim8b mRNA in 54 hours old zebrafish embryos incubated for 16 hours in presence or absence of R-roscovitine, a p53 activator in human and zebrafish cells. (TIF) [file pone.0046642.s007.tif]

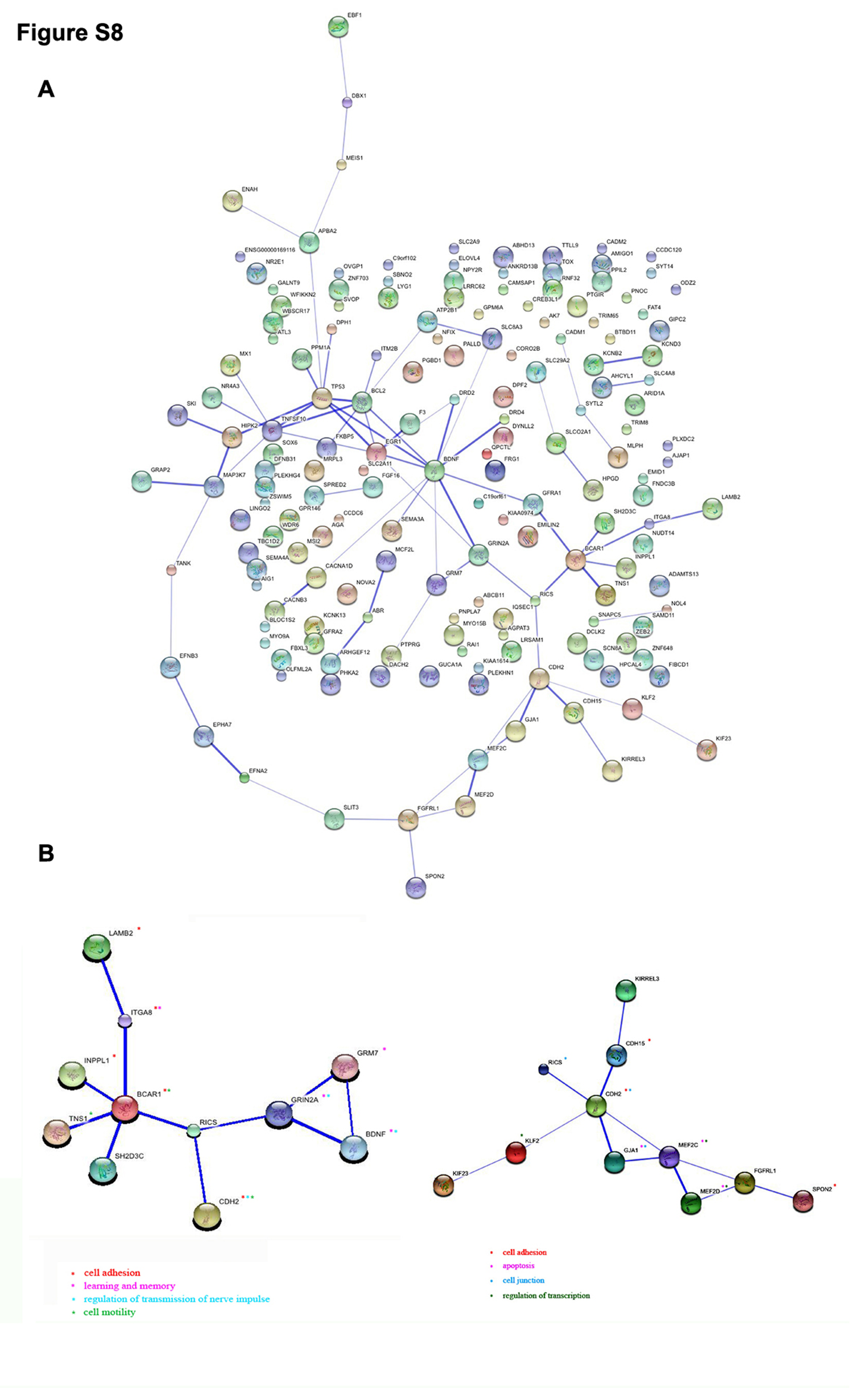

Supplement: Figure S8 — Protein networks of human orthologs of genes parasitized by EnSpm-N6_DR elements. (A) Global protein network of the human orthologs of genes colonized by EnSpm-N6_DR elements in zebrafish. (B) Additional protein subnetworks of these human orthologs. Asterisks' colors pinpoint the involvement of each gene to GO term-defined pathways. All subnetworks are visualized in STRING confidence view (the color saturation of the edges represents the confidence score of a functional association). (TIF) [file pone.0046642.s008.tif]
